# Upconversion nanoparticles doped optical lens: let’s see the near-infrared light

**DOI:** 10.1186/s12951-024-02564-8

**Published:** 2024-06-13

**Authors:** Yulin Hu, Baoqi Xu, Wei Li, Lin Liang, Fan Fei, Quankui Lin

**Affiliations:** National Engineering Research Center of Ophthalmology and Optometry, School of Biomedical Engineering, School of Ophthalmology and Optometry, Eye Hospital, Wenzhou Medical University, Wenzhou, 325027 China

**Keywords:** Near-infrared, Optical lens materials, Up conversion nanoparticles, Visualization

## Abstract

The human cannot detect light with a wavelength exceeding 700 nm, primarily due to limitations in the physiological structure of the human eye. However, in certain specific scenarios, the ability to detect near-infrared (NIR) light proves to be extremely valuable. To attain this desired capability, NIR up conversion nanoparticles (UCNPs) were prepared and doped in the optical lens materials, aiming to obtain a NIR light “visible” optical lens. It is demonstrated that the doping of UCNPs in the optical lens materials does not significantly impact on their mechanical properties, optical properties, surface properties and it exhibits excellent biocompatibility in cell and animal experiments. More importantly, the UCNPs doping can convert NIR light into visible light within the material effectively and stably. The eyes can “see” the NIR light after wearing such UCNPs doped optical lens. Such NIR light visible optical lens could have great potential in actual applications.

**Graphical Abstract:**

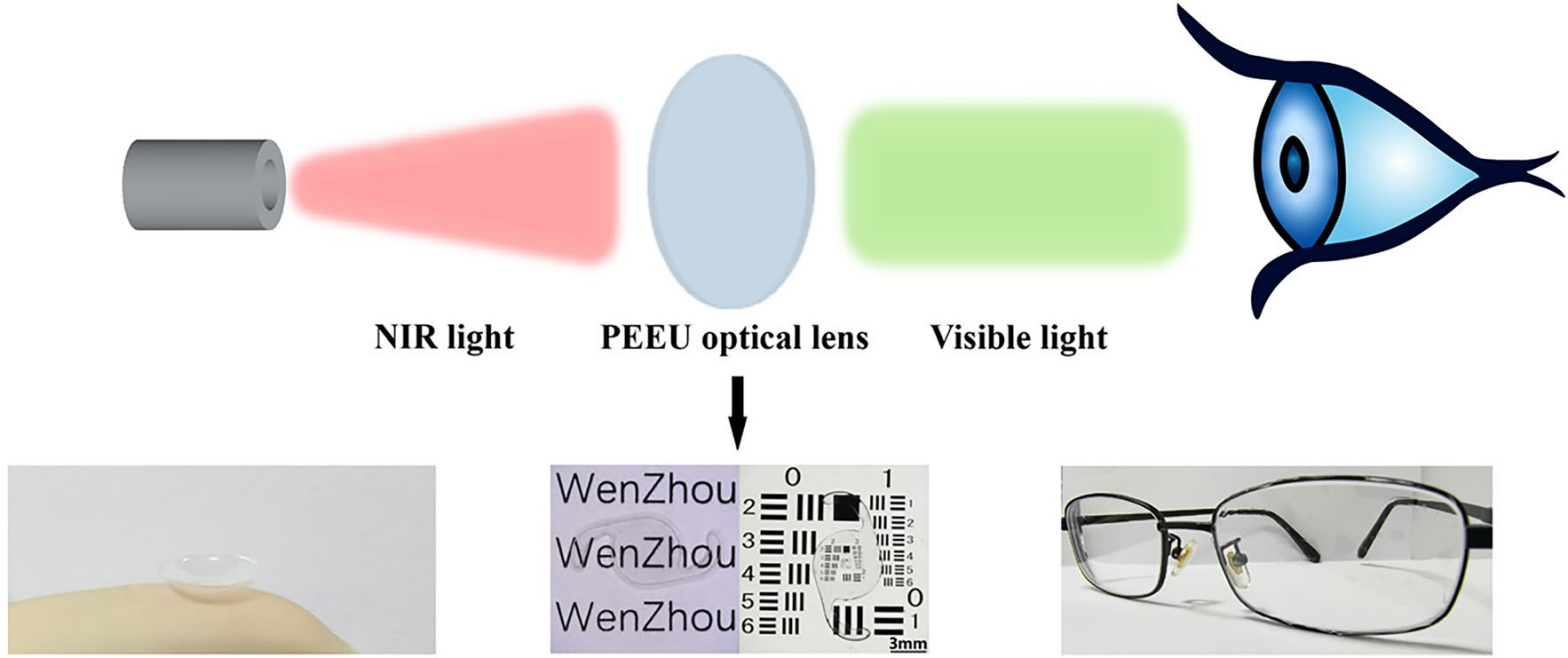

## Introduction

Vision is an extremely important way for humans to perceive the outside world. Yet the human eye can only detect light in the visible light spectrum between 380 and 780 nm [1, 2]. However, to “see” the longer wavelength light, such as near-infrared (NIR) light, has significant importance in daily life, scientific research, military affairs, and so on. For example, “Seeing” the NIR light can help drivers see potential hazards such as pedestrians, animals, and obstacles in the dark during night driving. This can greatly reduce the risk of accidents and improve road safety [3, 4]. More importantly, “Seeing” the NIR light will facilitate the military personnel to gather intelligence, conduct reconnaissance, and perform operations in low light conditions [5]. In addition, it can also be used in wildlife observation, search and rescue operations, and astronomy [6, 7]. Overall, the ability to “see” the NIR light has significant practical and scientific implications.

Upconversion nanoparticles (UCNPs) are a type of nanomaterial that can convert low-energy photons into higher-energy ones. This unique property makes them attractive for a variety of applications such as imaging, sensing, and photovoltaics [8–10]. Due to their low toxicity and biocompatibility, UCNPs have also been applied in the in vivo biomedical fields [11–14].UCNPs are typically made of rare-earth ions embedded in a host matrix, and their size can range from a few nanometers to tens of nanometers. When excited by NIR light radiation, UCNPs can emit visible or ultraviolet light. That is, it can converse the NIR light into visible or ultraviolet light.

**Scheme 1:**
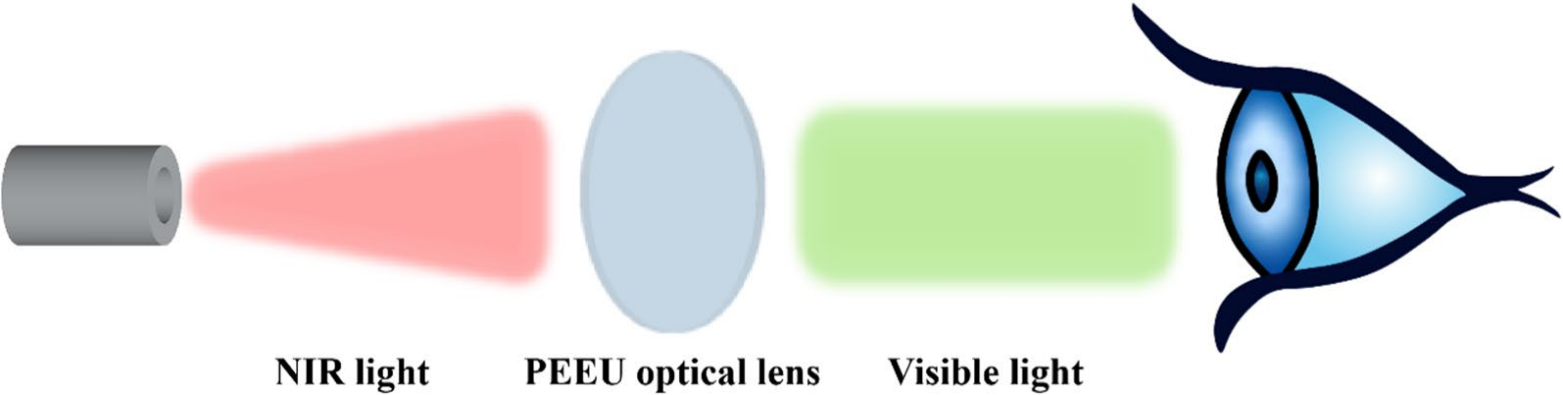
Schematic diagram of the PEEU optical lens converts NIR light into visible light

In our previous investigations, we have designed and obtained various functional optical lenses via surface modification, new bulk materials synthesis as well as nanoparticle doping in the bulk materials [15–18]. Especially, we have synthesized a new copolymer materials Poly (ethylene glycol phenyl ether methacrylate-*co*-2-(2-ethoxyethoxy)ethyl acrylate) (PEE), which is feasible for optical lens application [15]. Herein, we introduced UCNPs into the PEE optical lens materials to fabricate a UCNPs doped PEE optical lens (PEE-UCNPs, PEEU). It is assumed that when NIR light passes through, the upconversion ability of UCNPs will convert NIR light into visible light in the optical lens, achieving the purpose of NIR light visualization. This material provides a feasible method for breaking through the human visual threshold and has certain practical value in actual applications (Scheme 1).

## Experimental section

### Materials

Erbium chloride (ErCl_3_, 99.9%), Gadolinium chloride (GdCl_3_, 99.9%), Ytterbium chloride (YbCl_3_, 99.9%), Sodium chloride (NaCl), Ammonium fluoride (NH_4_F), Ethylene glycol, 2-(2-ethoxyethoxy) ethyl acrylate (EA), 2, 2’-Azobis(2-methylpropionitrile) (AIBN) were purchased from Aladdin (Shanghai, China). Polyethylene glycol 4000 (PEG4000) and ethylene glycol phenyl ether methacrylate (EGPEMA) were purchased from Macklin (Shanghai, China). Poly (ethylene glycol) diacrylate (PEGDA) was purchased from Sigma-Aldrich. All chemicals were of analytical grade and used following the manufacturers’ instructions. Cell culture medium DMEM/F12 (1:1), fetal bovine serum (FBS), trypsin, and penicillin–streptomycin were purchased from Gibco. Phosphate-buffered saline (PBS) was purchased from Boster Biotechnology. Cell counter kit-8 (CCK-8) and Calcein/PI Cell Viability/ Cytotoxicity Assay Kit were obtained from Beyotime Biotechnology. Most of the chemical reagents were used without further purification except polymerization monomers, which were purified by the inhibitor remover.

### Materials synthesis and preparation

*980 nm-excited UCNPs preparation*: NaGdF_4_:Yb/Er UCNPs was synthesized by an improved one-step hydrothermal method [19, 20]. 2.4 mmol NaCl, 0.96 mmol GdCl_3_, 0.216 mmol YbCl_3_, 0.024 mmol ErCl_3_, and 5 mg PEG4000 were dissolved in 20 mL ethylene glycol, followed with 15 mL 6 mmol NH_4_F in ethylene glycol solution adding and stirred for 30 min. Then, the mixed solution was transferred to stainless-lined autoclave and reacted in an oven at 200 ℃ for 12 h. The product was collected by centrifugation (10,000 rpm, 10 min), washed with ethanol and ultrapure water three times and dried at 60 °C. Thus, the NaGdF_4_:Yb^3+^, Er^3+^ UCNPs were prepared.

*PEEU Synthesis*: The synthesis of PEEU optical lens materials were similar with the synthesis of PEE optical lens materials except that the UCNPs were added when the reaction monomers mixture were prepared [15]. Briefly, Pretreated EGPEMA and EA were mixed at a mass ratio of 7:3, and then added the crosslinker PEGDA (1 wt%), initiators AIBN (0.5 wt%) and a certain amount of UCNPs. Ultrasonication was carried out for 10 min to ensure a well-dispersion of the mixed solution. Next, the reaction mixture was magnetically stirred for 30 min and bubbled with a stream of nitrogen (N_2_) for 30 min to remove the oxygen (O_2_). The degassed reaction mixture was then injected into an optical lens mold and polymerized for 24 h at 65 ℃. Finally, the obtained materials were washed by alcohol and ultra-pure water sequentially. Thus, the PEEU optical lens materials were obtained. For different NIR light conversion efficiency assay, different UCNPs contents, including none (0 wt%), 0.2 wt% or 0.4 wt% UCNPs doped PEEU materials were prepared, and abbreviated as PEE (0 wt%), PEEU (0.2 wt%), PEEU (0.4 wt%).

### Characterization process

Malvern Laser Particle Sizer (Malvern Instrument Ltd., Malvern, UK) measurement was used to measure the hydrated particle size and potential size of UCNPs. Fluorescence spectrophotometer (SpectraMax 190, USA) was used to test the fluorescence properties of UCNPs, using a 980 nm laser instead of a xenon lamp as the light source. The water contact angle and wettability were evaluated by contact angle analyzer (Data Physics Instrument GmbH, OCA25). Standard dumbbell-shaped specimens were used to test the mechanical properties, and the tensile test was carried out at the rate of 10 mm/min at 25 ℃ (CJinan Metis Test Technology, MT2503). The optical transmittances of the materials were measured by ultraviolet-visible spectrophotometer (UV-Vis) (Shimadzu, Japan) in the wavelength range of 400–800 nm with air as the reference. The refractive index of the materials was measured by Abbe refractometer (NAR-1 T, ATAGO CO., LTD, JAPAN). The lamp source used in all the experiments was NIR laser 980 nm (Lasever Inc, LSR980NL-3W-FC).

### NIR light conversion effect evaluation

As mentioned above, different UCNPs contents doped PEEU optical lens materials, including PEE (0 wt%), PEEU (0.2 wt%), PEEU (0.4 wt%) were synthesized for different NIR light conversion efficiency assay. The different optical lens materials were placed in dark environment, and illuminated by the 980 nm NIR light at different intensities. Photographs were taken and recorded to evaluate the conversion effect of NIR light.

### In vitro biocompatibility evaluation

The PEEU optical lens materials were made into intraocular lens disk (Φ = 6 mm) and soaked in 300 μL of PBS (pH = 7.4) for 24 h to obtain the leaching solution. Human lens epithelial cells (HLECs) and human corneal epithelial cells (HCECs) were cultured in DMEM/F12 (1:1) medium supplemented with 10% FBS and 1% penicillin–streptomycin at 37 ℃, 5% CO_2_ humidified atmosphere. Cells were seeded in the 96 well tissue culture polystyrene plates at density of 8000 cells per well. After incubated for 24 h, 20 μL material leachate solutions were added and incubation for another 24 h. Then the cells were incubated with 10% CCK-8 working solution for 2 h, and the OD value was measured by a microplate reader to calculate cell viability. The fluorescent images of the cells were also taken after live/dead staining.

### Pupillary light reflex experiment

The animal experiments were carried out according to the National Institutes of Health Guidelines for the Care and Use of Laboratory Animals and approved by the experimental animal ethics committee of Wenzhou Medical University. Due to the similarity between the eye structure of rabbit and human, it is widely used in the study of ophthalmology and pathology [21]. Moreover, contrast sensitivity has now become a common indicator for assessing the human visual system’s ability to process spatial frequency information, and some studies have indicated that the rabbit’s contrast sensitivity is significantly lower than that of humans [22]. Therefore, the same light that has a significant stimulating effect on the rabbit’s eye will also have a significant stimulating effect on the human eye. Finally, in order to explore whether rabbits can “see” NIR light by PEEU optical lens, pupillary light reflex (PLR) experiments were carried out. Three New Zealand white rabbits with weight about 2.5–3 kg were used in this study. The PEE and PEEU (0.4 wt%) optical lenses were prepared for use. On the other hand, the rabbits were dark-adapted for 1 h. Then, illuminate the rabbit eye fitted with PEEU or PEE optical lenses with 980 nm NIR light, and the pupil responses were observed by eye images taken as well as the diameter and area of pupil calculation. The light intensity was 1 W/cm^2^, with a distance of around 15 cm, and the irradiation area was circular, concentrated in the center of the eye.

### Subcutaneous implantation experiment

The animal experiments were carried out according to the National Institutes of Health Guidelines for the Care and Use of Laboratory Animals and approved by the experimental animal ethics committee of Wenzhou Medical University. Two New Zealand white rabbits with weight about 2.5–3 kg were selected in this study. The PEEU optical lens materials were sterilized by ethylene oxide disinfection. After anesthetizing, the rabbits’ side abdomens were shaved and disinfected with iodophor solution. Three small skin incisions were made and the materials were implanted. After subcutaneous implantation, the incisions were sutured. On the 7th day postoperatively, the rabbits were humanely sacrificed under anesthesia. The adjacent tissue around the implants were isolated, fixed in a paraformaldehyde solution, and stained with hematoxylin − eosin (H&E) for histological observation.

### Statistical analysis

Three parallel samples were set in the experiments, and the results were expressed as mean ± standard deviation. One-way analysis of variance was adopted to compare the statistical difference between two groups. P < 0.05 (*), P < 0.01 (**), P < 0.001 (***) represent significant differences, and P > 0.05 represent no significance difference (ns).

## Results and discussion

### Synthesis of UCNPs and PEEU

**Fig. 1:**
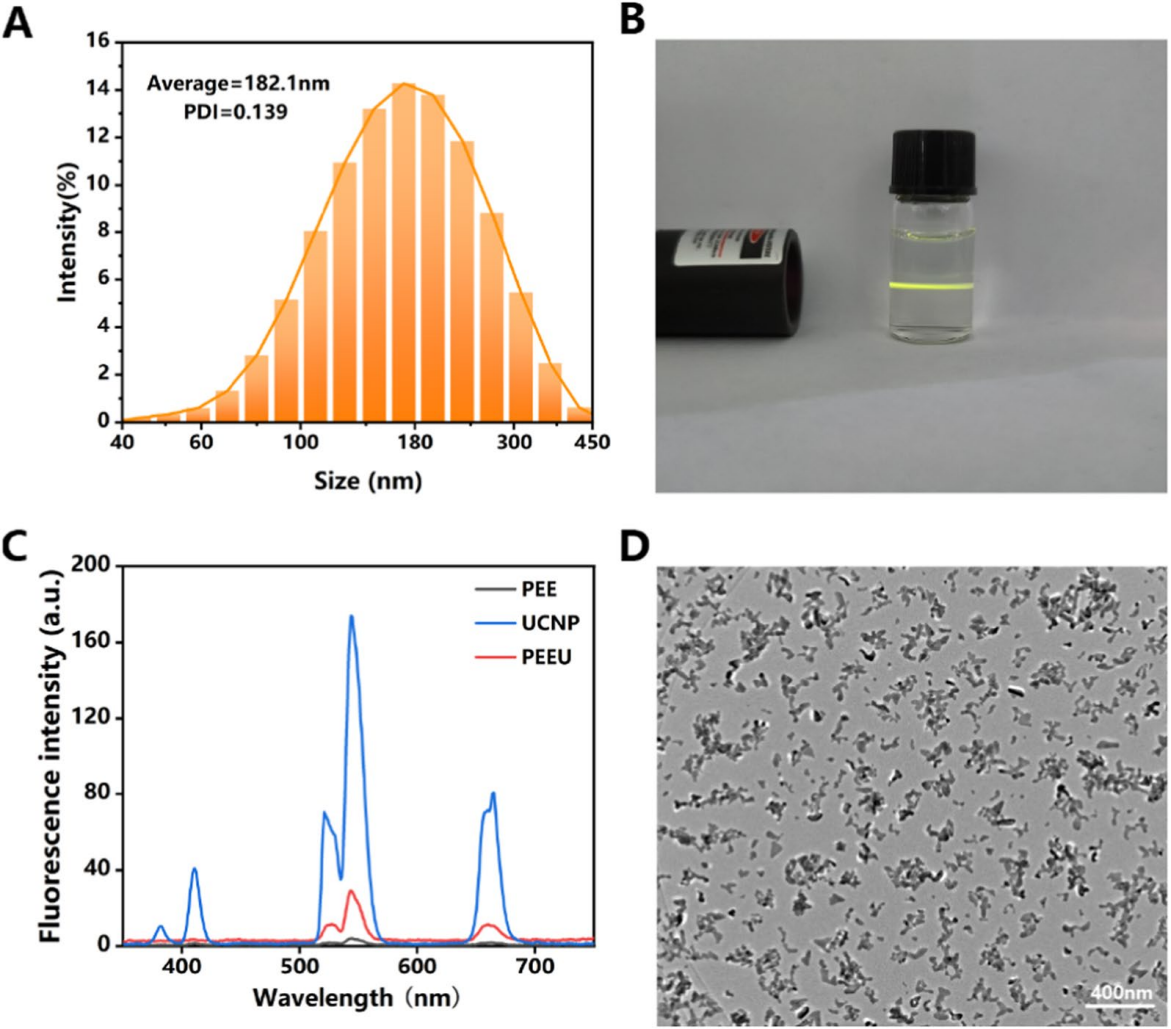
**A** Particle size of UCNPs. **B** Direct view of 1 mg/ml UCNPs aqueous solution irradiated by 980 nm laser. **C** Emission spectrum of PEE, PEEU and UCNPs upon 980 nm laser excitation. **D** TEM image of UCNPs

Figure 1A shows the hydrated particle size of the prepared UCNPs. A wide distribution with mean size approximate 157 nm was observed. When a beam of naked eye invisible 980 nm NIR light passed through the 1 mg/ml UCNPs aqueous solution, a beam of chartreuse light was visible in the solutions (Fig. 1B). This result demonstrates the UCNPs can effectively convert the invisible NIR light into the visible chartreuse light. The upconversion function of the UCNPs was further confirmed by the fluorescence emission spectra. As shown in Fig. 1C, peaks at wavelengths of 383, 412, 521, 545, and 664 nm appear in the fluorescence emission spectrum when it was excited by the 980 nm NIR light, which were all located in the visible light wavelength range. It can also be seen that almost no notable emission peaks was found in the PEE optical lens materials (Fig. 1C). However, the characteristic fluorescence emission peaks of UCNPs appeared in the PEEU optical lens materials, which not only demonstrated the successful doping of the UCNPs in the PPE materials, but also indicated that the synthesized PEEU materials may also have effective upconversion functions. As indicated by TEM (Fig. 1D), it can be seen that the prepared UCNPs are of a branched structure composed of aggregated crystallites, with clearly visible interfacial lines between the crystal faces, indicating that it has good crystallinity.

### Physicochemical properties

**Fig. 2:**
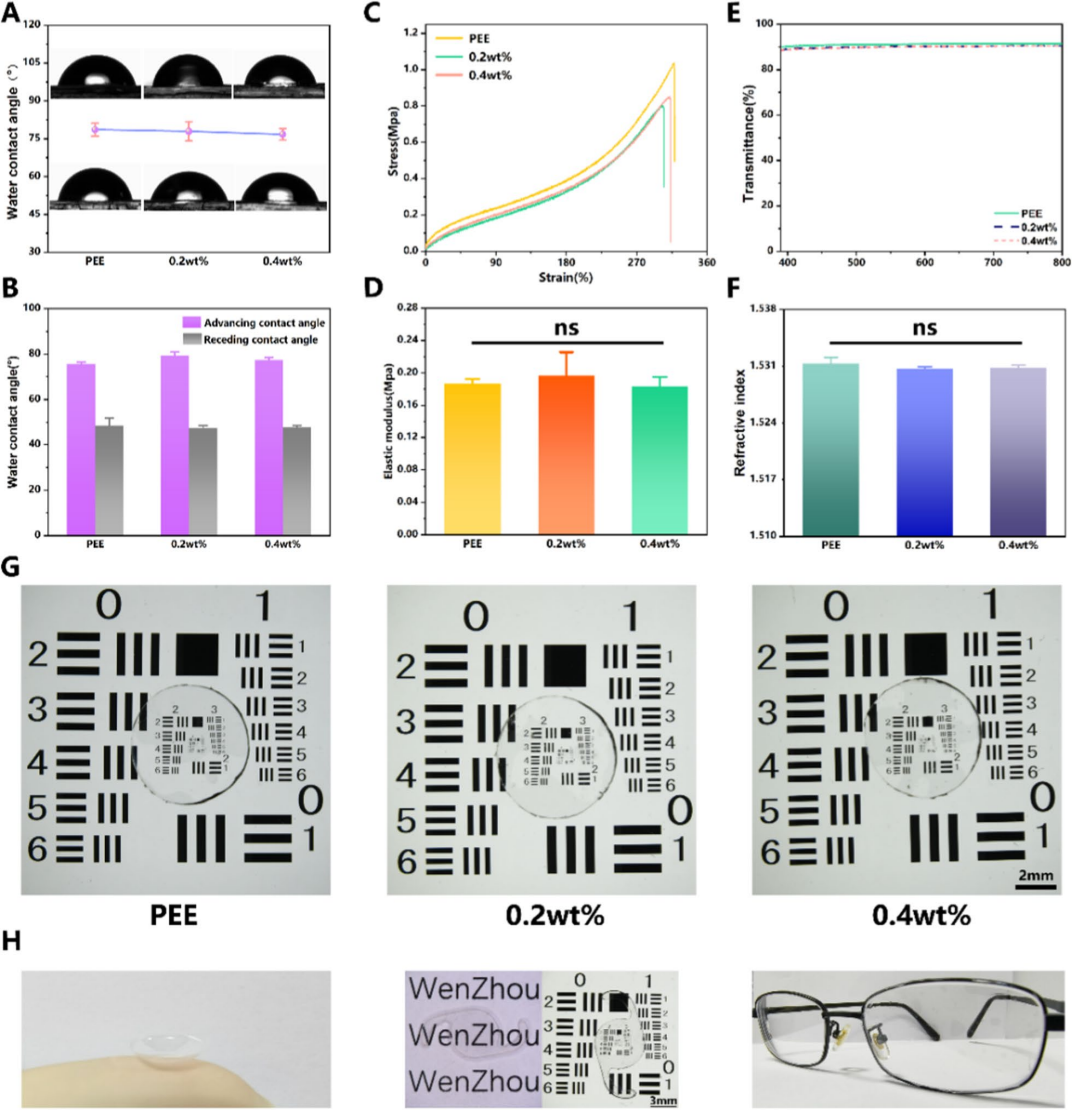
Physicochemical properties of PEE (0 wt%), PEEU (0.2 wt%), PEEU (0.4 wt%) (**A**) Static water contact angle. **B** Dynamic water contact angle. **C** Light transmittance. **D** Elastic modulus. **E** Light transmittance. **F** Refractive index. **G** Representative image of the optical lens materials placed on an optical resolution plate. **H** The image of CL, IOL, and coating film for glasses lenses made of PEEU

As an optical lens material, the physicochemical properties of PEEU should also be concerned. The surface wettability of the materials was evaluated by the contact angle between the water droplets and each type of material. Figure 2A and B showed the surface static contact angle and the dynamic contact angle of the optical lens materials. It can be observed that the water contact angles of each group were close to 77°. The dynamic contact angle can better reflect the wettability of the material, and the advancing contact angle and receding contact angle of each group are both around 77° and 47° respectively. The mechanical tensile tests were conducted to obtain the tensile stress–strain curves of samples, and the elastic modulus were calculated from the curves. As shown in Fig. 2C, the representative stress–strain curves of the PEE (0 wt%), PEEU (0.2 wt%), PEEU (0.4 wt%) were essentially similar. Therefore, after statistical analysis, there was no significant difference in the elastic modulus among these materials. No matter the UCNPs doped or not, all the elastic modulus of different samples were around 0.18 Mpa (Fig. 2D). This result indicated that the materials kept low elastic modulus even if the UCNPs were doped, which was still feasible for optical lens manufacture, such as contact lens (CL), intraocular lens (IOL) [15]. The optical performances of these materials were also investigated. As seen in Fig. 2E and F, the transmittances of these materials were all around 90%, and the refractive indexes were approximately 1.53. There was not significant difference between the PEE materials and after different contents of UCNPs doping in the optical performances. Furthermore, the imaging qualities of the PEEU materials were also investigated by the USAF1951 resolution chart. The optical lens materials were placed on the resolution charts and the photos were taken by the stereomicroscope (Fig. 2G). It can be seen that the image has high clarity, with sharp imaging details in all of these optical lens materials. No apparent distortion, and other interference factor was observed. The doping or not, and doping amount of the UCNPs does not significant change in the image quality of the optical lens materials. In conclusion, the particle loading has no significant impact on various aspects of the optical lens performance. The PEEU materials were then manufactured into the different kinds of optical lenses, such as CL, IOL, and coating film for glasses lenses (Fig. 2H). It is easy for manufacturing and the optical appearance was good. It indicated that such PEEU materials have significant feasibility in optical lenses.

### NIR light conversion effect experiment

**Fig. 3:**
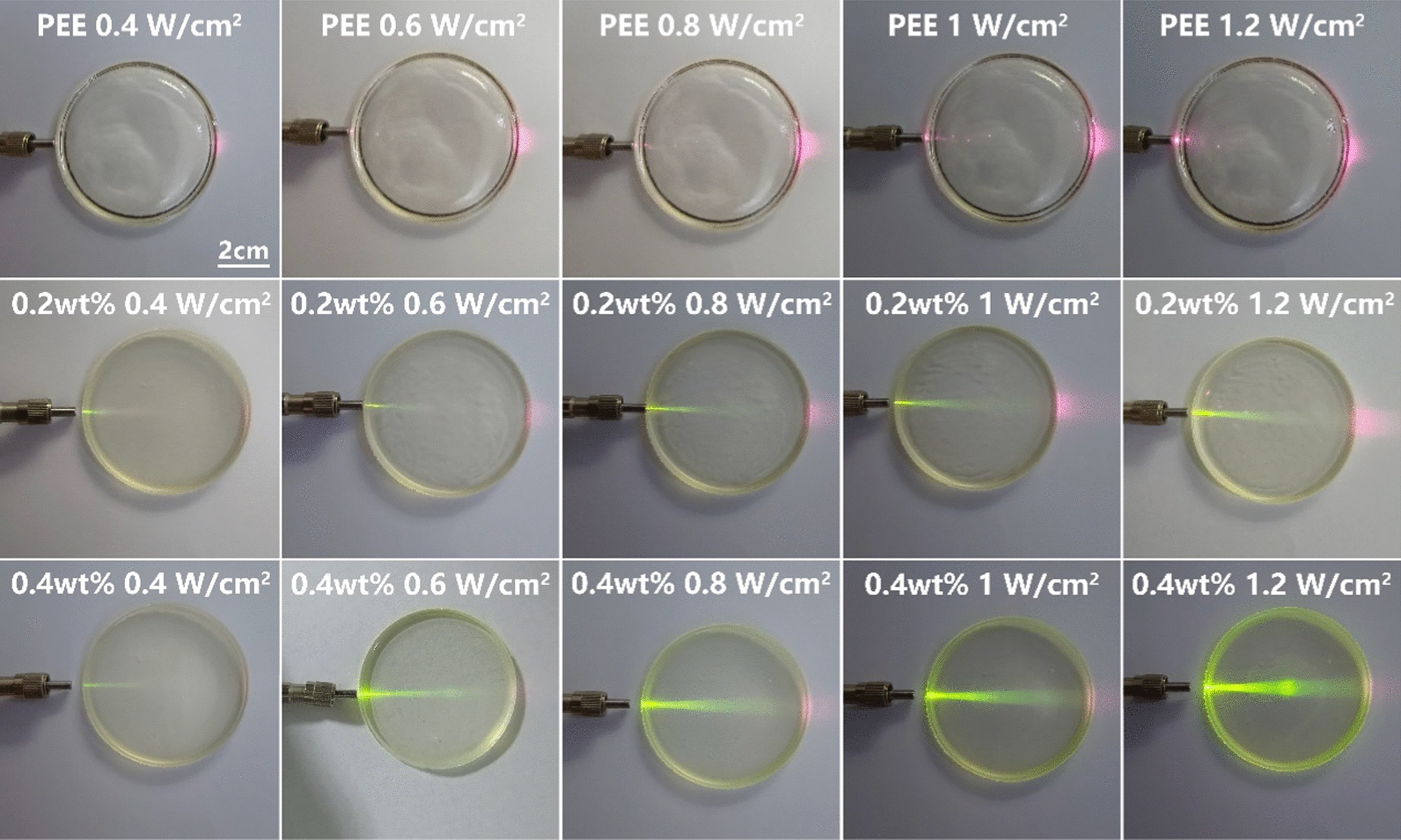
Direct view of the spectacle lenses with different UCNPs contents doping (0 wt%, 0.2 wt%, 0.4 wt%) under 980 nm laser irradiation at different intensities

The optical lens materials were made into circular plain spectacle lenses with diameter of 6.3 cm. For easy observation, the 980 nm NIR light was illuminated through the side of the PEEU spectacle lenses. Different intensities of the NIR lights were acted and light conversion in the spectacle lenses were evaluated the intensity of visible chartreuse light beams directly. As shown in Fig. 3, there was no visible light observed in the PEE spectacle lenses, no matter what intensity of the NIR light was illuminated through them. It should be noted that the pink light out of the spectacle lenses is due to the scatter effect of the photograph by the camera, there was no pink light detected when observe by the naked human eyes. After UCNPs doping, the light upconversion was observed. It can be seen that even in a low NIR light illumination intensity (0.4 W/cm^2^), the visible chartreuse light beams appeared, no matter in PEEU (0.2 wt%) or PEEU (0.4 wt%) spectacle lenses. It is evident that the intensity of the chartreuse light conversion significantly increased with the increase of UCNPs concentration in the PEEU spectacle lenses. Thus, the PEEU (0.4 wt%) optical lens materials were used because of its significant NIR light conversion effect in the subsequent in vitro and in vivo experiments. It can also be concluded that as the intensity of NIR light increased, the intensity of the converted chartreuse light also increased, showing a positive correlation. These results demonstrated the chartreuse NIR light conversion effect of PEEU and indicated that the conversion effect can be adjusted by modulated the proportion of doped UCNPs, and the intensity of NIR light can be roughly estimated by visible light intensity [2].

### In vitro cell culture experiment

**Fig. 4:**
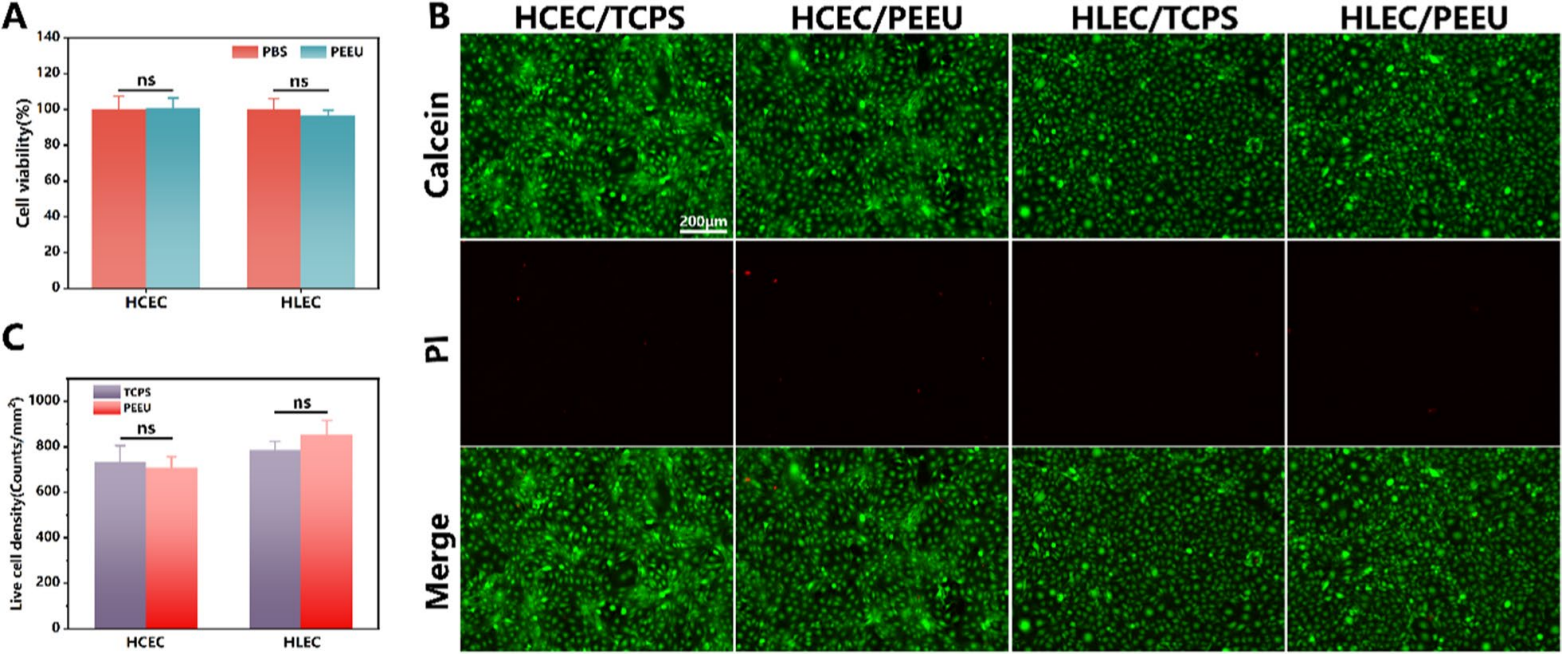
**A** Cell viabilities of the PEEU optical lens material leaching solution and PBS. **B** Representative fluorescent images of cells after different treatment. **C** Quantification of the living cell density after different treatment

As mentioned above, the PEEU optical lens materials can be manufactured not only as spectacle lenses, but also as CL or IOL [15]. Thus, the biocompatibility should also be investigated. The cytotoxicity was evaluated by co-culturing the material leaching solution with cells. Compared with the control group (TCPS), there was no significant difference observed among the groups, indicating that the PEEU optical lens materials had little impact on cell viability (Fig. 4A). Then, the cell morphology was observed through live-dead staining, in which green fluorescence representing living cells and red fluorescence representing dead cells. From Fig. 4B we can see that the images basically showed green fluorescence, indicating normal cell morphology and good growth. The cell numbers were also quantified by ImageJ software (National Institutes of Health, 1.53 t). Figure 4C shows that there was no significant difference in cell numbers between the PEEU optical lens materials and TCPS. These results demonstrated that PEEU optical lens materials have good biocompatibility and no significant cell toxicity.

### Pupillary light reflex experiment

**Fig. 5:**
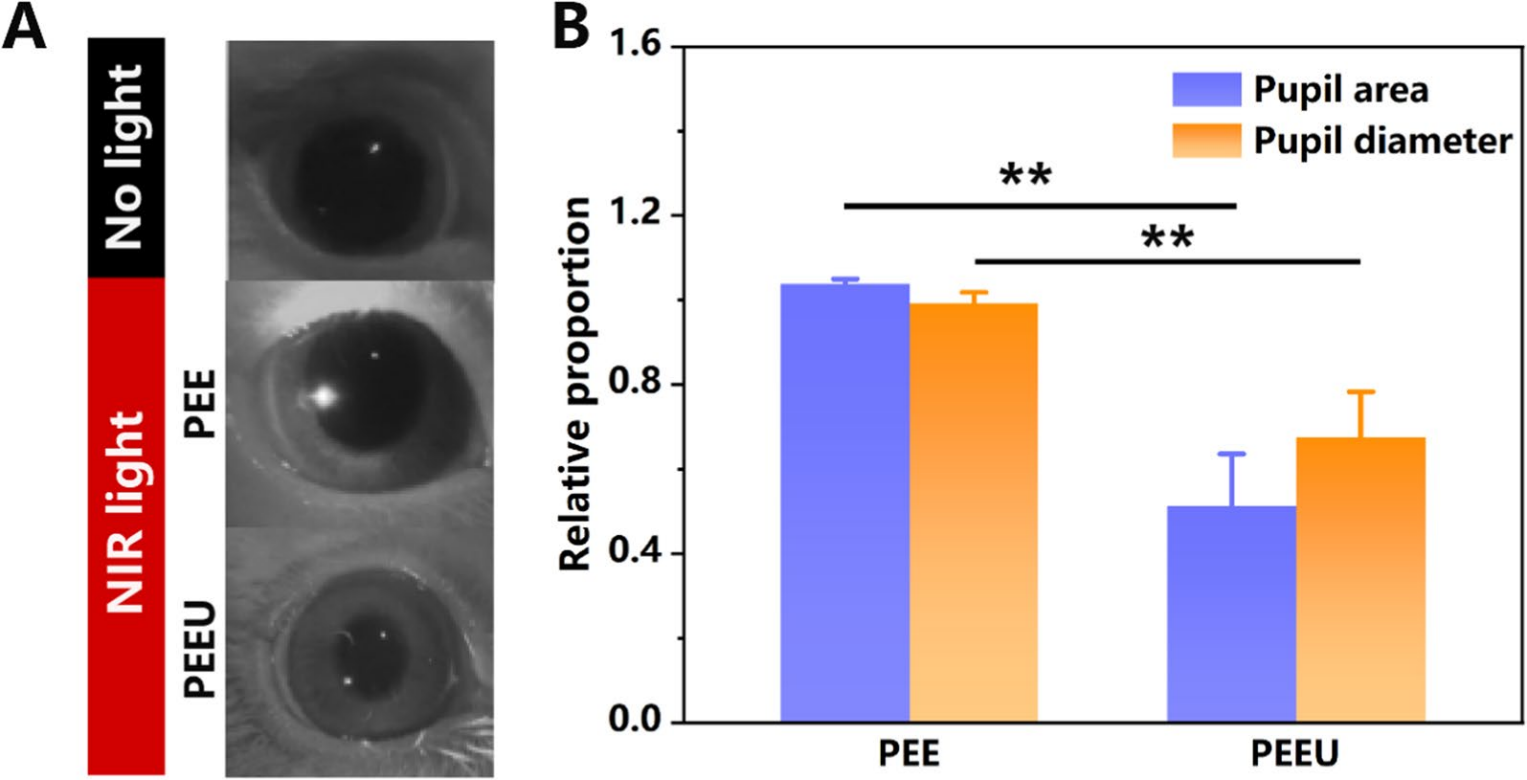
**A** The image of the rabbit eye under different conditions: after dark adaptation, worn PEE or PEEU optical lenses and stimulated by 980 nm NIR light.** B** Pupil diameter and area in the different optical lens worn eyes stimulated by 980 nm NIR light

To reveal whether PEEU optical lens can help eyes “see” the NIR light, the in vivo PLR experiments were carried out and the results were shown in Fig. 5A. There were no apparent changes in pupil size compared to dark adaptation (No light group) when the 980 nm laser was stimulated to the PEE optical lens worn rabbit’s eyes. However, a significant change in pupil size was obtained after stimulation in the PEEU optical lens worn rabbit eyes. The area and diameter of the pupil images were then calculated and analyzed, with the pupil after dark adapted as the reference. The results were shown in Fig. 5B. It was evident that the area and diameter of the pupils in the PEE optical lens worn eyes were close to 1, indicating that the pupil parameters in PEE optical lens eyes were essentially the same as they were in dark adaptation. In contrast, the pupil diameter and area in PEEU optical lens worn eyes exhibited a significant reduction and a notable difference compared with they were after dark adaption. The pupil reflex to light may be treated as a feedback process to control the light impinging upon the retina. Thus, the pupil response directly influents the eyes see the light or not. Therefore, these results collectively demonstrated that the PEEU optical lens possesses up-conversion capabilities, effectively converting NIR light into visible light that can be detected by the eyes. In practical usage scenarios, the convergence degree and intensity of NIR light may decrease relatively, which could lead to a weaker conversion effect, and considering that temperature changes, humidity, dust, and other factors could all affect the final usage effect. However, this study mainly aims to demonstrate that when these nanoparticles are combined with optical materials, they can possess the ability of upconversion, thereby providing a feasible approach for the visualization of NIR light.

### Subcutaneous implantation experiment

**Fig. 6:**
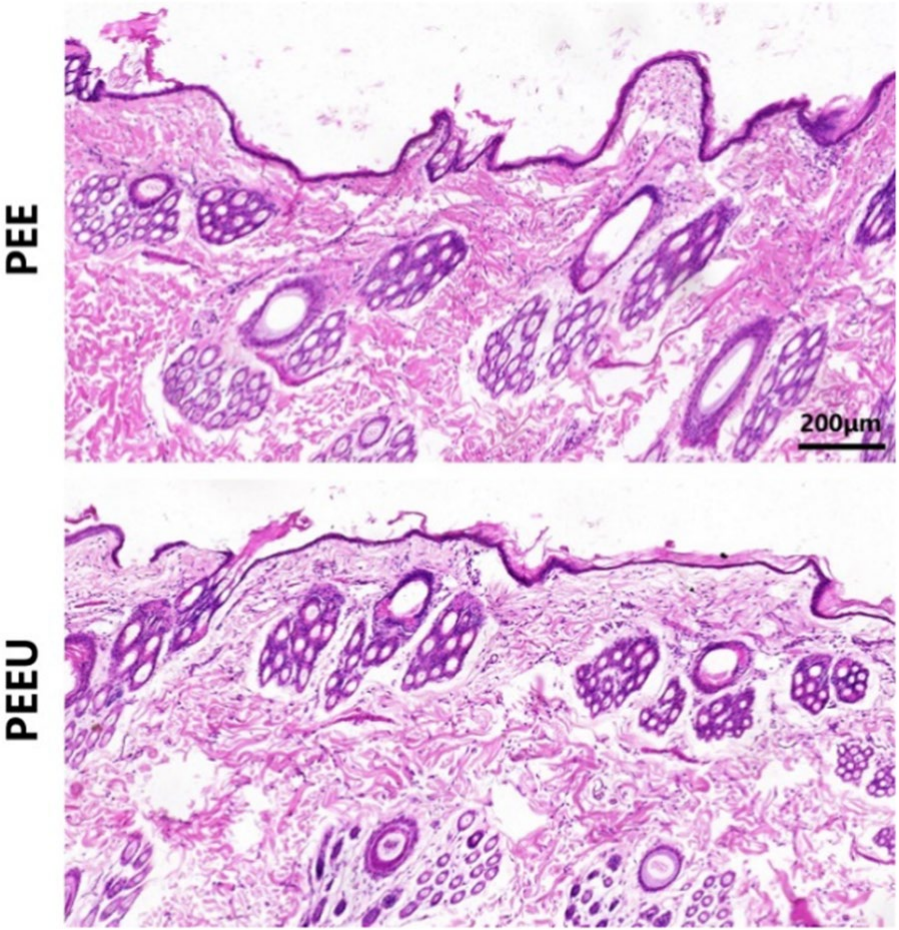
Microscopic images of tissue sections with H&E staining of PEE and PEEU optical lens after subcutaneous implantation

The in vivo biocompatibility of the PEEU optical lens was also investigated. The materials were implanting subcutaneously in rabbits for a week, followed by the retrieval of surrounding tissues for sectioning and staining. As shown in Fig. 6, the tissue sections revealed no significant inflammatory cells in both PEE and PEEU optical lenses. Among all the implants, the connective tissue (stained pink) displayed clear tissue boundaries. There were no inflammatory cells, necrotic cells, macrophage infiltration, foreign body giant cells, or activated fibroblasts found in the slices, revealing that the materials have good in vivo biocompatibility.

## Conclusion

To “see” the NIR light of human beings has significant importance in daily life, scientific research, or military affairs. In this study, a NIR upconversion nanoparticle which can convert NIR light into visible light was prepared and doped into our optimized PEE optical lens materials when synthesis, obtaining upconversion nanoparticle doped organic inorganic hybrid materials PEEU. The in vitro investigations have demonstrated that the upconversion nanoparticle doping does not change their modulus, wettability, biocompatibility, as well as the optical properties, including the transmittance, reflective index or imaging quality. However, the introduction of the upconversion nanoparticle gives the synthesized optical lens NIR light conversion effect, which converse the 980 nm NIR light into different wavelengths of visible lights (the visible blend color is chartreuse). More importantly, when the PEEU materials were made into optical lenses and worn on the rabbit eyes, they can “see” the invisible NIR light, as their pupil responses were acting significant difference. Thus, the fabricated PEEU optical lens can effectively realize the visualization of NIR lights. In summary, a NIR light visible optical lens materials were obtained by doping the NIR upconversion nanoparticle in the PEE materials. It can also be supposed that it can help eyes to “see” different wavelengths of NIR light or other lone wavelengths un-visible lights. Thus, such NIR light visible optical lens has considerable potential in various fields such as civilian, security, and military applications.

## Data Availability

No datasets were generated or analysed during the current study.
